# Elevated glucose level leads to rapid COVID-19 progression and high fatality

**DOI:** 10.1186/s12890-021-01413-w

**Published:** 2021-02-24

**Authors:** Wenjun Wang, Mingwang Shen, Yusha Tao, Christopher K. Fairley, Qin Zhong, Zongren Li, Hui Chen, Jason J. Ong, Dawei Zhang, Kai Zhang, Ning Xing, Huayuan Guo, Enqiang Qin, Xizhou Guan, Feifei Yang, Sibing Zhang, Lei Zhang, Kunlun He

**Affiliations:** 1 Key Laboratory of Ministry of Industry and Information Technology of Biomedical Engineering and Translational Medicine, Chinese PLA General Hospital, Beijing, 100853 People’s Republic of China; 2 Translational Medical Research Center, Chinese PLA General Hospital, Beijing, 100853 People’s Republic of China; 3 Medical Artificial Intelligence Research Center, Chinese PLA General Hospital, Beijing, 100853 People’s Republic of China; 4 China-Australia Joint Research Center for Infectious Diseases, School of Public Health, Xi’an Jiaotong University Health Science Center, Xi’an, Shanxi 710061 People’s Republic of China; 5 Central Clinical School, Faculty of Medicine, Nursing and Health Sciences, Monash University, Melbourne, VIC Australia; 6 Department of Medical Information, Huoshenshan Hospital, Wuhan, Hubei People’s Republic of China; 7 Department of Medical Information, The 940th Hospital of PLA Joint Logistics Support Force, Lanzhou, People’s Republic of China; 8 Department of Infectious Disease, the Fifth Medical Center, Chinese PLA General Hospital, Beijing, 100039 People’s Republic of China; 9 Department of Medical Administration, Chinese PLA General Hospital, Beijing, 100853 People’s Republic of China; 10 Department of Radiology, Chinese PLA General Hospital, Beijing, 100853 People’s Republic of China; 11 Department of Pulmonary and Critical Care Medicine, Chinese PLA General Hospital, Beijing, 100853 People’s Republic of China; 12 Melbourne Sexual Health Centre, Alfred Health, Melbourne, Australia

**Keywords:** COVID-19, Progression, Fatality, Risk factors

## Abstract

**Objectives:**

We aimed to identify high-risk factors for disease progression and fatality for coronavirus disease 2019 (COVID-19) patients.

**Methods:**

We enrolled 2433 COVID-19 patients and used LASSO regression and multivariable cause-specific Cox proportional hazard models to identify the risk factors for disease progression and fatality.

**Results:**

The median time for progression from mild-to-moderate, moderate-to-severe, severe-to-critical, and critical-to-death were 3.0 (interquartile range: 1.8–5.5), 3.0 (1.0–7.0), 3.0 (1.0–8.0), and 6.5 (4.0–16.3) days, respectively. Among 1,758 mild or moderate patients at admission, 474 (27.0%) progressed to a severe or critical stage. Age above 60 years, elevated levels of blood glucose, respiratory rate, fever, chest tightness, c-reaction protein, lactate dehydrogenase, direct bilirubin, and low albumin and lymphocyte count were significant risk factors for progression. Of 675 severe or critical patients at admission, 41 (6.1%) died. Age above 74 years, elevated levels of blood glucose, fibrinogen and creatine kinase-MB, and low plateleta count were significant risk factors for fatality. Patients with elevated blood glucose level were 58% more likely to progress and 3.22 times more likely to die of COVID-19.

**Conclusions:**

Older age, elevated glucose level, and clinical indicators related to systemic inflammatory responses and multiple organ failures, predict both the disease progression and the fatality of COVID-19 patients.

## Introduction

In December 2019, an outbreak of novel coronavirus pneumonia (COVID-19) caused by SARS-CoV-2 was reported in Wuhan city, China. Since then, COVID-19 had rapidly spread to more than 17.6 million cases, with over 680,000 deaths worldwide as of August 2, 2020 [1]. A total of 84,428 cases and 4,634 deaths were reported in China, and approximately 81% of cases and 97% of deaths were from Wuhan city of August 2, 2020 [2]. As the hardest-hit city by the COVID-19 pandemic, Wuhan initiated a metropolitan-wide quarantine on January 23, 2020, which terminated all public transportation in the city and intercity links. The quarantine lasted for 76 days and was eventually lifted on April 8, 2020 [3–6]. The metropolitan-wide quarantine approach had since become a common practice to combat the COVID-19 epidemic globally.

At the peak of the epidemic, Wuhan authorities constructed an emergency hospital specialized in treating people infected with SARS-CoV-2, inspired by the prefab hospital erected in Beijing during the 2003 SARS outbreak. This emergency hospital, Huoshenshan hospital, was constructed within ten days and the first hospital specialized in treating patients with COVID-19 in the world [7–9]. This hospital closed on April 15, 2020, one week after the metropolitan-wide quarantine was lifted [10]. During the 73 days of operation, the hospital treated 3,059 patients with COVID-19, of whom 2,961 recovered [7].

Identifying the risk factors of COVID-19 disease progression and fatality provides important evidence to support appropriate clinical management and optimize medical resource allocation [11–13]. The risk factors for COVID-19 progression include comorbidities with chronic diseases (hypertension, diabetes, cardiovascular disease and liver disease), old age, low lymphocyte and albumin counts and elevated levels of lactate dehydrogenase, c-reactive protein, red blood cell distribution width, blood urea nitrogen and direct bilirubin [14]. In particular, patients with old age, neutrophilia, thrombocytopenia, higher lactate dehydrogenase and d-dimer levels were more likely to develop Acute Respiratory Distress Syndrome (ARDS) [15, 16]. Further, older age, d-dimer level greater than 1 µg/mL and a high Sequential Organ Failure Assessment score on admission would contribute to a higher in-hospital fatality of COVID-19 patients [17]. Liang et al. [18], based on chest radiography abnormality and nine clinical indicators were able to predict the risk of developing critical illness with an area-under-the-curve of 0.88. A systematic review and meta-analysis examine risk factors associated with adverse clinical outcomes in patients with COVID-19 [19]. However, most of these studies were limited by their relatively small sample sizes, and many patients had not progressed to the study endpoints by the time the study was conducted, leading to bias and unreliable prediction for disease progression and fatality. Besides, some analyses of risk factors were not adjusted for potential confounding effects, leading to false associations.

In this study, we retrospectively collected the complete hospitalization information from 2,433 patients who were admitted to Huoshenshan hospital during its 73 days of operation. We explored on the time for disease progression among patients in various disease stages at admission and determined their risk of disease progression. We also identified clinical risk factors that predict the COVID-19 disease progression and fatality among these patients.

## Methods

### Study design and patients

We established a retrospective observational study cohort, based on 3,059 cases admitted to the Huoshenshan hospital in Wuhan between February 4 and April 15, 2020. The exclusion criteria were: (1) Patients who were not confirmed by a positive result of severe acute respiratory syndrome coronavirus 2 detection in respiratory specimens by the reverse transcriptase polymerase chain reaction assay, or in serum by the specific IgM and IgG antibody detection; (2) Patients who referred to other medical institution during hospitalization; (3) Patients who were admitted to the hospital multiple times; (4) Patients were younger than 18 years old; (5) Patients without laboratory data included in this study within the 24 h after admission. This study tracked the progression of COVID-19 patients from admission until one of the endpoints (discharged or death).

### Clinical and outcome indicators

Demographic, clinical, laboratory, treatment, and clinical outcome data were obtained from the hospital’s electronic clinical medical records. At the first clinical consultation, demographic, clinical and laboratory data were collected within the first day after admission. Treatment data and clinical outcomes (including the event of disease progression, time of each disease stages, fatality, duration of hospitalization and endpoint status) were also collected during the course from admission to the study endpoints. We defined the event of disease progression as a mild or moderate patient at admission would progress to severe or critical stage at the first time during hospitalization.

### Clinical definitions

The severity of COVID-19 was defined according to the Guidance 7th edition [20]. Patients were classified as ‘mild’ if there was no evidence of pneumonia on imaging nor any of the features for moderate or higher severity; as ‘moderate’ if they had evidence of pneumonia on imaging but no features of severe or higher severity; as ‘severe’ if they meet any of the following criteria: (1) respiratory distress (≥ 30 breaths/min); (2) oxygen saturation ≤ 93% at rest on room air; (3) arterial partial pressure of oxygen (PaO2) or fraction of inspired oxygen (FiO2) ≦ 300 mmHg (l mmHg = 0.133 kPa); and as ‘critical’ if they required mechanical ventilation, had a septic shock or required admission to ICU. Comorbidities were defined according to ICD10-CM code [21]. Detailed definitions for clinical symptoms were provided in the supplemental materials. We considered a patient progressing to a severe or critical disease stage when the individual had none of the severe or critical stages at admission but developed these stages for the first time during hospitalization.

Patients had to meet all the following criteria before being discharged: (1) body temperature returned to normal (< 37.5 °C) for three consecutive days; (2) respiratory symptoms improved substantially; (3) pulmonary imaging showed an obvious absorption of inflammation; and (4) two consecutive negative nuclei acid tests, each at least 24 h apart.

### Statistical analysis

We presented continuous variables as the median and interquartile range (IQR) and examined the differences between disease severity groups using the Kruskal–Wallis one-way ANOVA We presented categorical variables with the corresponding percentage and examined the differences using χ^2^ test or Fisher’s exact test. We conducted survival analyses on disease progression and fatality based on a competing risk framework. The outcome variables included: (1) the event of the first time disease progression to severe or critical disease states among mild or moderate patients at admission, and (2) in-hospital fatality among patients with severe or critical at admission. Discharge from the hospital was considered as a competing risk event. Five clinical indicators (interleukin-6, natriuretic peptide type B, supersensitive troponin I, myoglobin and procalcitonin.) with more than 30% of entries missing were excluded from the analysis. Data imputation was performed if missing percentage < 30% using Multivariate Imputation by Chained Equations. Statistically significant variables in the univariate analysis were ranked and further selected using LASSO regression [22]. The number of variables was defined as the number of variables when λ = λmin in LASSO, or the total number of events divided by 10 (event per variable > 10 rule)[23], whichever is smaller. Variables, which particularly reported in previous literatures were included in final analysis. The pooled set of variables were then included for the final multivariable cause-specific Cox proportional hazard model. Cumulative incidence curves were plotted to demonstrate the incidence of differences between different risk levels of key variables. A *p* value of < 0.05 was considered statistically significant. Statistical analyses were conducted using the R software (version 3.6.1).

## Results

### Demographic characteristics of patients

After excluding 214 patients who were only diagnosed clinically according to the Guidance 7th edition [20], 46 patients who referred to other medical institution, 16 patients who were admitted to the hospital multiple times, six patients were younger than 18 years old. Further, we excluded 328 patients without laboratory data included in this study within the 24 h after admission, we included 2,433 COVID-19 patients in the final analysis. Fifty patients died during hospitalization, and 2,383 were discharged, corresponding to a case-fatality ratio of 2.1%. Patient’s median age was 60.0 years (IQR 50.0–68.0), and 50.2% were male (Table 1). The most common symptoms or signs on admission were cough (55.7%), fatigue (38.9%), and shortness of breath (25.0%). Hypertension being the most common comorbidity (31.6%), followed by diabetes (14.3%) and coronary heart disease (6.7%). During hospitalization, 847 (34.8%) patients received antibiotics, 1233 (50.7%) received antivirals, and 68 (2.8%) received non-invasive mechanical ventilation and 42 (1.7%) received invasive mechanical ventilation.

**Table 1:** Basic demographic characteristics, signs and symptoms, comorbidities, laboratory findings, treatment and clinical outcomes of 2,433 COVID-19 patients admitted to the Huoshenshan hospital

Variable	All patients (N = 2,433)	Clinical classification at admission				*p* value
		Mild (N = 25)	Moderate (N = 1,733)	Severe (N = 635)	Critical (N = 40)	
*Demographic characteristics*						
Age (IQR)—year	60.0 (50.0, 68.0)	36.0 (29.0, 54.0)	58.0 (48.0, 66.0)	65.0 (56.0, 72.0)	67.0 (58.0, 80.3)	< 0.001**
Male gender—no. (%)	1222 (50.2)	14 (56.0)	891 (51.4)	301 (47.4)	16 (40.0)	0.171
Smoking history—no. (%)	205 (8.4)	3 (12.0)	145 (8.4)	52 (8.2)	5 (12.5)	0.592
Drinking history—no. (%)	154 (6.3)	1 (4.0)	117 (6.8)	32 (5.0)	4 (10.0)	0.291
Respiratory rate > 20 (%)	689 (28.3)	4 (16.0)	426 (24.6)	232 (36.5)	27 (67.5)	< 0.001**
Pulse rate > 100 (%)	356 (14.6)	3 (12.0)	233 (13.4)	106 (16.7)	14 (35.0)	0.001*
Systolic blood pressure ≥ 140 (%)	655 (26.9)	6 (24.0)	433 (25.0)	203 (32.0)	13 (32.5)	0.007*
Diastolic blood pressure ≥ 90 (%)	562 (23.1)	3 (12.0)	414 (23.9)	139 (21.9)	6 (15.0)	0.227
*Signs and symptoms—no. (%)*						
Body temperature (IQR)—°C	36.5 (36.3, 36.7)	36.5 (36.3, 36.7)	36.5 (36.3, 36.7)	36.5 (36.3, 36.7)	36.5 (36.3, 36.8)	0.022*
Fever (temperature ≥ 37.5 °C)	63 (2.6)	0 (0.0)	34 (2.0)	27 (4.3)	2 (5.0)	0.012*
Cough	1356 (55.7)	3 (12.0)	969 (55.9)	363 (57.2)	21 (52.5)	< 0.001**
Fatigue	947 (38.9)	4 (16.0)	677 (39.1)	251 (39.5)	15 (37.5)	0.116
Diarrhea	65 (2.7)	2 (8.0)	44 (2.5)	18 (2.8)	1 (2.5)	0.288
Chest tightness	292 (12.0)	0 (0.0)	190 (11.0)	97 (15.3)	5 (12.5)	0.007*
Shortness of breath	608 (25.0)	0 (0.0)	411 (23.7)	189 (29.8)	8 (20.0)	< 0.001**
*Comorbidities—no. (%)*						
Hypertension	769 (31.6)	5 (20.0)	497 (28.7)	252 (39.7)	15 (37.5)	< 0.001**
Diabetes	349 (14.3)	0 (0.0)	233 (13.4)	109 (17.2)	7 (17.5)	0.013*
Coronary heart disease	163 (6.7)	1 (4.0)	94 (5.4)	64 (10.1)	4 (10.0)	< 0.001**
Cancer	42 (1.7)	0 (0.0)	29 (1.7)	12 (1.9)	1 (2.5)	0.739
Chronic bronchitis	53 (2.2)	0 (0.0)	30 (1.7)	19 (3.0)	4 (10.0)	0.009*
Cerebrovascular disease	89 (3.7)	0 (0.0)	48 (2.8)	37 (5.8)	4 (10.0)	< 0.001**
Chronic kidney disease	43 (1.8)	0 (0.0)	23 (1.3)	17 (2.7)	3 (7.5)	0.011*
Chronic obstructive pulmonary disease	19 (0.8)	0 (0.0)	9 (0.5)	9 (1.4)	1 (2.5)	0.059*
Hepatitis	23 (0.9)	1 (4.0)	19 (1.1)	3 (0.5)	0 (0.0)	0.189
*Laboratory findings (IQR)*						
C-reactive protein (mg/L)	2.1 (0.8, 7.4)	0.5 (0.2, 2.5)	1.8 (0.7, 5.3)	3.4 (1.2, 14.8)	45.0 (8.7, 104.0)	< 0.001**
D-dimer (mg/L)	0.4 (0.2, 0.8)	0.2 (0.2, 0.3)	0.3 (0.2, 0.6)	0.6 (0.3, 1.2)	1.6 (1.0, 4.1)	< 0.001**
Lactate dehydrogenase (IU/L)	175.0 (150.3, 211.7)	150.7 (128.9, 181.3)	169.1 (147.2, 199.7)	193.8 (163.7, 241.4)	332.3 (250.7, 430.9)	0.002*
White blood cell count (10^9^/L)	5.7 (4.7, 7.0)	5.7 (4.8, 7.0)	5.6 (4.7, 6.8)	5.8 (4.7, 7.2)	9.3 (7.5, 14.3)	< 0.001**
Lymphocyte count (10^9^/L)	1.5 (1.1, 1.9)	1.7 (1.5, 2.0)	1.5 (1.2, 1.9)	1.4 (0.9, 1.7)	0.8 (0.5, 1.3)	< 0.001**
Neutrophils count (10^9^/L)	3.5 (2.7, 4.6)	3.4 (2.5, 4.2)	3.4 (2.6, 4.4)	3.7 (2.8, 5.1)	8.4 (5.6, 13.5)	< 0.001**
Monocyte count (10^9^/L)	0.4 (0.3, 0.5)	0.4 (0.3, 0.5)	0.4 (0.3, 0.5)	0.4 (0.3, 0.6)	0.4 (0.2, 0.6)	< 0.001**
Basophils count (10^8^/L)	0.2 (0.1, 0.3)	0.2 (0.1, 0.3)	0.2 (0.1, 0.3)	0.2 (0.1, 0.3)	0.0 (0.0, 0.3)	< 0.001**
Eosinophils count (10^8^/L)	1.1 (0.6, 1.8)	1.3 (0.9, 1.8)	1.1 (0.6, 1.8)	1.1 (0.5, 1.9)	0.2 (0.0, 1.1)	< 0.001**
Prothrombin time (s)	12.8 (12.2, 13.5)	12.8 (12.3, 13.3)	12.8 (12.2, 13.4)	12.9 (12.3, 13.7)	14.1 (12.6, 16.6)	0.256
Total bilirubin (*μ*mol/L)	9.6 (7.4, 12.5)	9.5 (7.3, 10.9)	9.5 (7.3, 12.4)	9.8 (7.7, 12.6)	11.3 (8.4, 16.1)	0.561
Direct bilirubin (μmol/L)	3.4 (2.5, 4.5)	3.4 (2.9, 3.9)	3.3 (2.5, 4.3)	3.5 (2.6, 4.8)	5.6 (3.5, 8.9)	< 0.001**
Albumin (g/L)	38.4 (35.5, 40.7)	41.1 (38.9, 43.9)	38.8 (36.1, 41.0)	37.1 (33.9, 39.5)	33.3 (29.1, 37.2)	< 0.001**
Alkaline phosphatase (IU/L)	69.7 (58.3, 84.5)	66.6 (57.7, 78.1)	68.6 (57.7, 83.8)	71.7 (59.7, 84.7)	92.9 (77.7, 114.3)	0.202
Fibrinogen (g/L)	3.0 (2.6, 3.4)	2.5 (2.4, 3.1)	3.0 (2.6, 3.3)	3.0 (2.7, 3.5)	3.0 (2.6, 3.8)	0.007*
Creatinine (μmol/L)	64.1 (54.7, 75.3)	64.6 (52.9, 79.5)	63.5 (54.7, 74.6)	65.4 (55.2, 77.2)	56.5 (49.5, 93.1)	0.032*
Creatine kinase (U/L)	51.2 (37.0, 73.2)	73.8 (54.7, 100.9)	52.1 (38.4, 72.6)	46.7 (33.2, 72.7)	57.4 (29.4, 152.4)	0.083*
Creatine kinase-MB (IU/L)	8.6 (6.9, 10.9)	8.6 (7.0, 10.4)	8.4 (6.9, 10.5)	8.9 (7.1, 11.5)	14.7 (12.1, 22.7)	0.002*
Blood glucose (mmol/L)	4.9 (4.5, 5.7)	4.7 (4.2, 4.9)	4.8 (4.4, 5.5)	5.1 (4.5, 6.0)	6.4 (5.2, 8.4)	< 0.001**
Urea nitrogen (mmol/L)	4.4 (3.6, 5.4)	3.7 (3.1, 4.9)	4.2 (3.6, 5.2)	4.6 (3.7, 6.0)	7.5 (5.2, 10.3)	< 0.001**
Cystatin C (mg/L)	0.9 (0.8, 1.1)	0.8 (0.7, 1.0)	0.9 (0.8, 1.0)	1.0 (0.9, 1.1)	1.2 (0.9, 1.7)	< 0.001**
Platelets count (10^9^/L)	220.0 (180.0, 271.0)	235.0 (202.0, 274.0)	220.0 (184.0, 271.0)	220.0 (170.0, 267.0)	195.0 (89.0, 290.0)	0.009*
Alanine aminotransferase (IU/L)	21.7 (14.2, 36.1)	14.9 (10.8, 35.3)	22.1 (14.3, 36.4)	21.3 (14.2, 35.0)	38.8 (16.1, 51.5)	0.647
Aspartate aminotransferase (IU/L)	19.5 (15.4, 26.1)	17.9 (13.5, 25.3)	19.1 (15.2, 25.3)	20.1 (15.7, 28.6)	31.2 (24.2, 48.4)	0.001*
*Treatment—no. (%)*						
Intravenous antibiotics	847 (34.8)	2 (8.0)	515 (29.7)	294 (46.3)	36 (90.0)	< 0.001**
Antivirus treatment	1233 (50.7)	11 (44.0)	809 (46.7)	392 (61.7)	21 (52.5)	< 0.001**
Traditional Chinese medicine	2348 (96.5)	24 (96.0)	1679 (96.9)	611 (96.2)	34 (85.0)	0.007*
Systemic glucocorticoids	424 (17.4)	3 (12.0)	202 (11.7)	193 (30.4)	26 (65.0)	< 0.001**
Intravenous immunoglobin	119 (4.9)	0 (0.0)	39 (2.3)	65 (10.2)	15 (37.5)	< 0.001**
Invasive mechanical ventilation	42 (1.7)	0 (0.0)	9 (0.5)	16 (2.5)	17 (42.5)	< 0.001**
Noninvasive mechanical ventilation	68 (2.8)	0 (0.0)	15 (0.9)	27 (4.3)	26 (65.0)	< 0.001**
Extracorporeal membrane oxygenation	3 (0.1)	0 (0.0)	1 (0.1)	0 (0.0)	2 (5.0)	0.001*
Continuous renal-replacement therapy	22 (0.9)	0 (0.0)	6 (0.3)	6 (0.9)	10 (25.0)	< 0.001**
Convalescence plasma therapy	111 (4.6)	0 (0.0)	55 (3.2)	51 (8.0)	5 (12.5)	< 0.001**
*Clinical outcomes—no. (%)*						
Discharged	2383 (97.9)	25 (100.0)	1724 (99.5)	614 (96.7)	20 (50.0)	< 0.001**
Deceased	50 (2.1)	0 (0.0)	9 (0.5)	21 (3.3)	20 (50.0)	< 0.001**

Compared with the clinical classification at admission, the *P value is between 0.05 and 0.001; the **p value < 0.001

### Clinical progression and regression during hospitalization

**Fig. 1:**
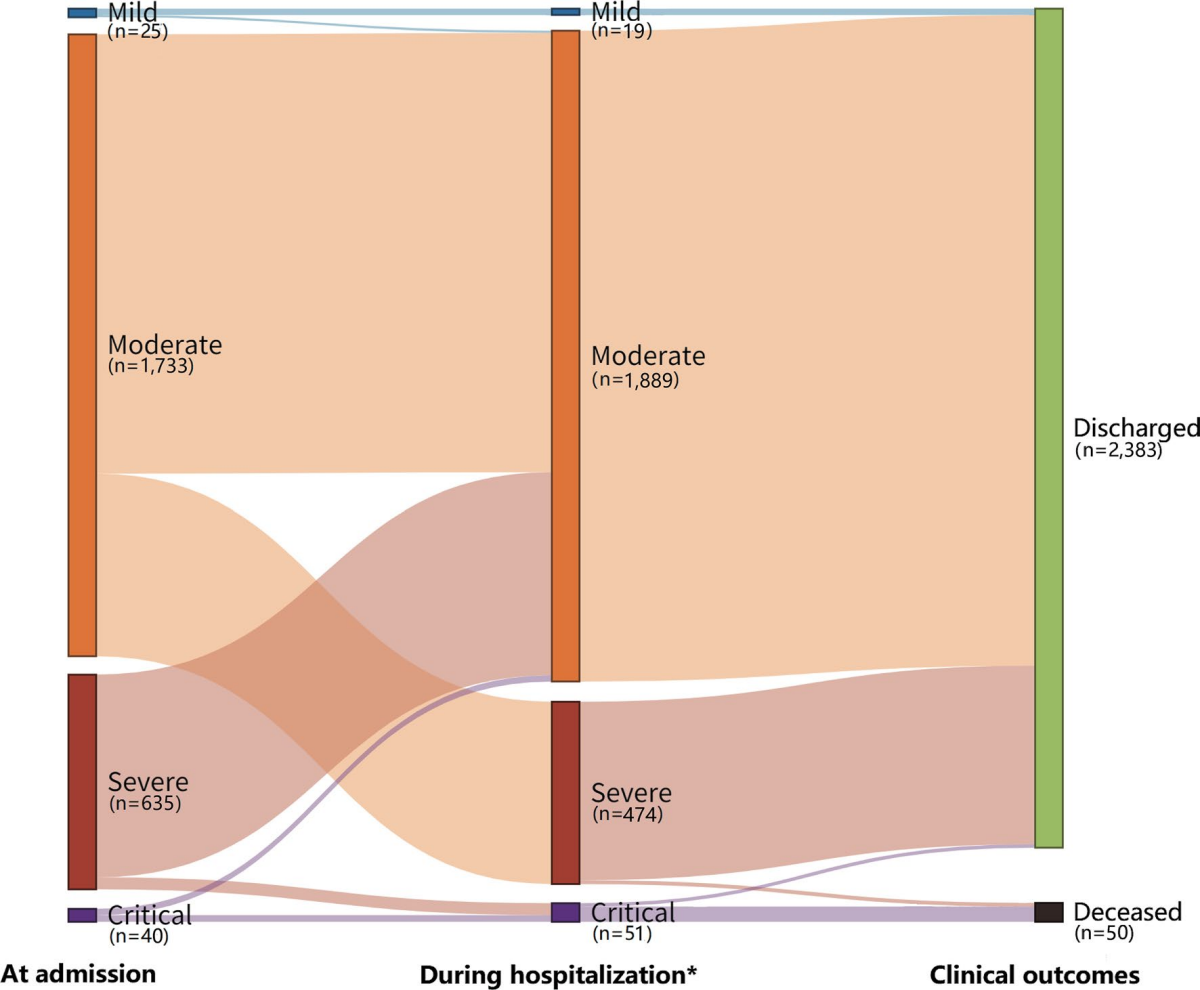
Sankey figure showing the proportion of patients in the COVID-19 disease progression at admission, during hospitalization and their clinical outcomes. (*COVID-19 patient’s first disease progression during hospitalization)

**Fig. 2:**
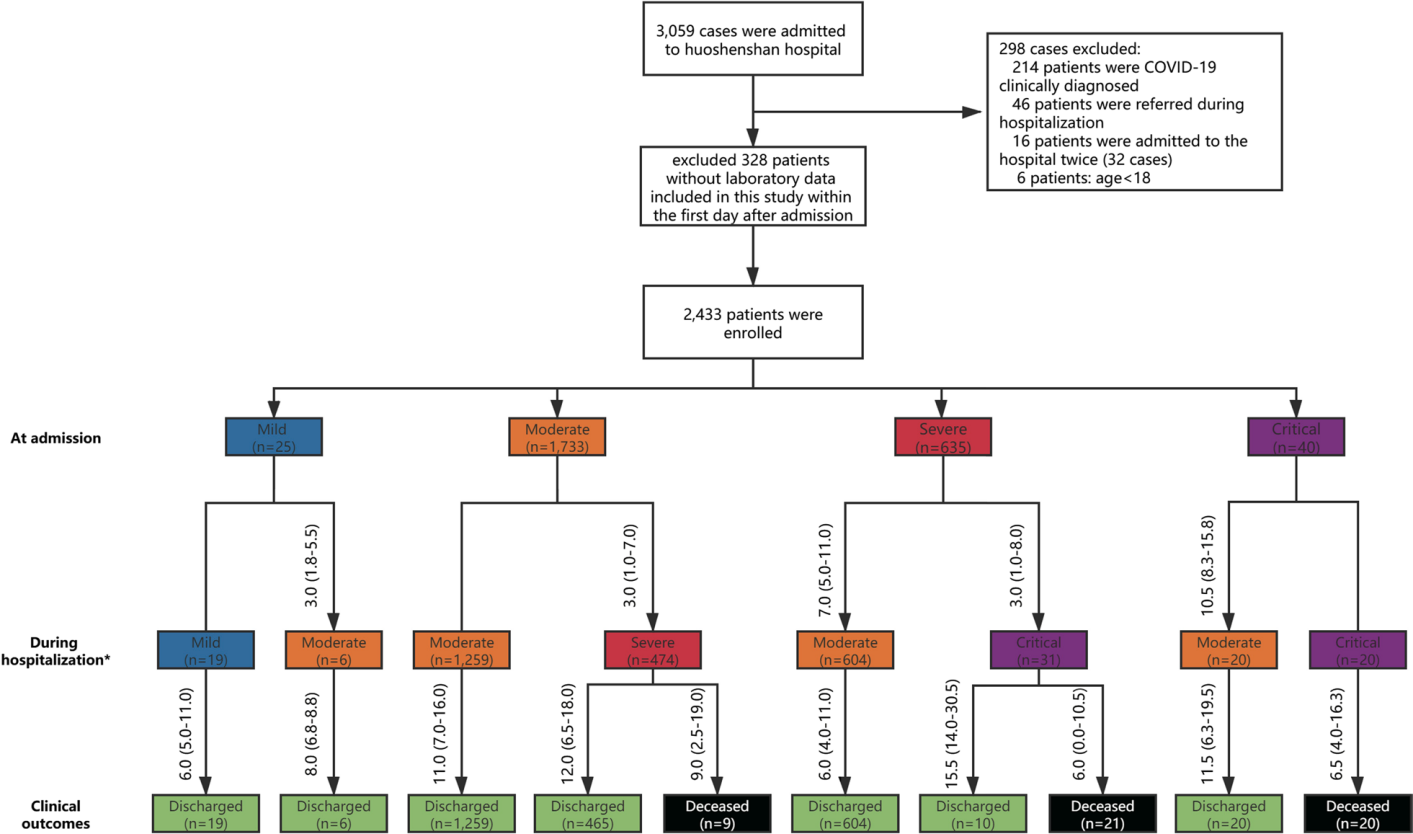
A flowchart showing the duration in days for disease progression and time to clinical endpoints in 2,433 COVID-19 patients at the Huoshenshan hospital. (*COVID-19 patient’s first disease progression during hospitalization)

Among 25 mild patients at admission, 19 retained mild and were discharged after 6.0 (5.0–11.0) days; six patients who progressed to moderate severity in 3.0 (1.8–5.5) days, but all discharged after another 8.0 (6.8–8.8) days. Of 1,733 moderate patients at admission, 1,259 patients retained moderate and discharged after 11.0 (7.0–16.0) days. In contrast, 474 patients progressed to the severe state in 3.0 (1.0–7.0) days, but all recovered and were discharged after another 12.0 (6.5–18.0) days, and 9 patients deceased after 9.0 (2.5–19.0) days. Of 635 severe patients at admission, 604 patients regressed to moderate severity in 7.0 (5.0–11.0) days and were discharged after another 6.0 (4.0–11.0) days. Thirty-one patients progressed to critical severity after 3.0 (1.8–8.0) days, and of whom only 10 patients were discharged after 15.5 (14.0–30.5) days, and 21 died after 6.0 (0.0–10.5) days. Of 40 critical patients at admission, 20 patients regressed to moderate severity after 10.5 (8.3–15.8) days and were discharged after another 11.5 (6.3–19.5) days, and the remaining 20 patients died after 6.5 (4.0–16.3) days (Figs. 1 and 2).

Across all patients, it required a median of 3.0 (1.8–5.5) days to progress from mild to moderate, 3.0 (1.0–7.0) days from moderate to severe, 3.0 (1.0–8.0) days from severe to critical and 6.5 (4.0–16.3) from critical to fatality. In contrast, it required 7.0 (5.0–11.0) days to regress from severe or critical to moderate severity and 6.5 (4.0–11.0) days from moderate to discharge. The median admission-to-discharge time among mild, moderate, severe and critical patients on admission were 7.0 (5.5–12.0), 13.0 (9.0–19.0), 15.0 (8.0–22.0), and 25.0 (15.3–34.8) days, respectively.

### Contributing factors to disease progression and COVID-19 fatality

**Fig. 3:**
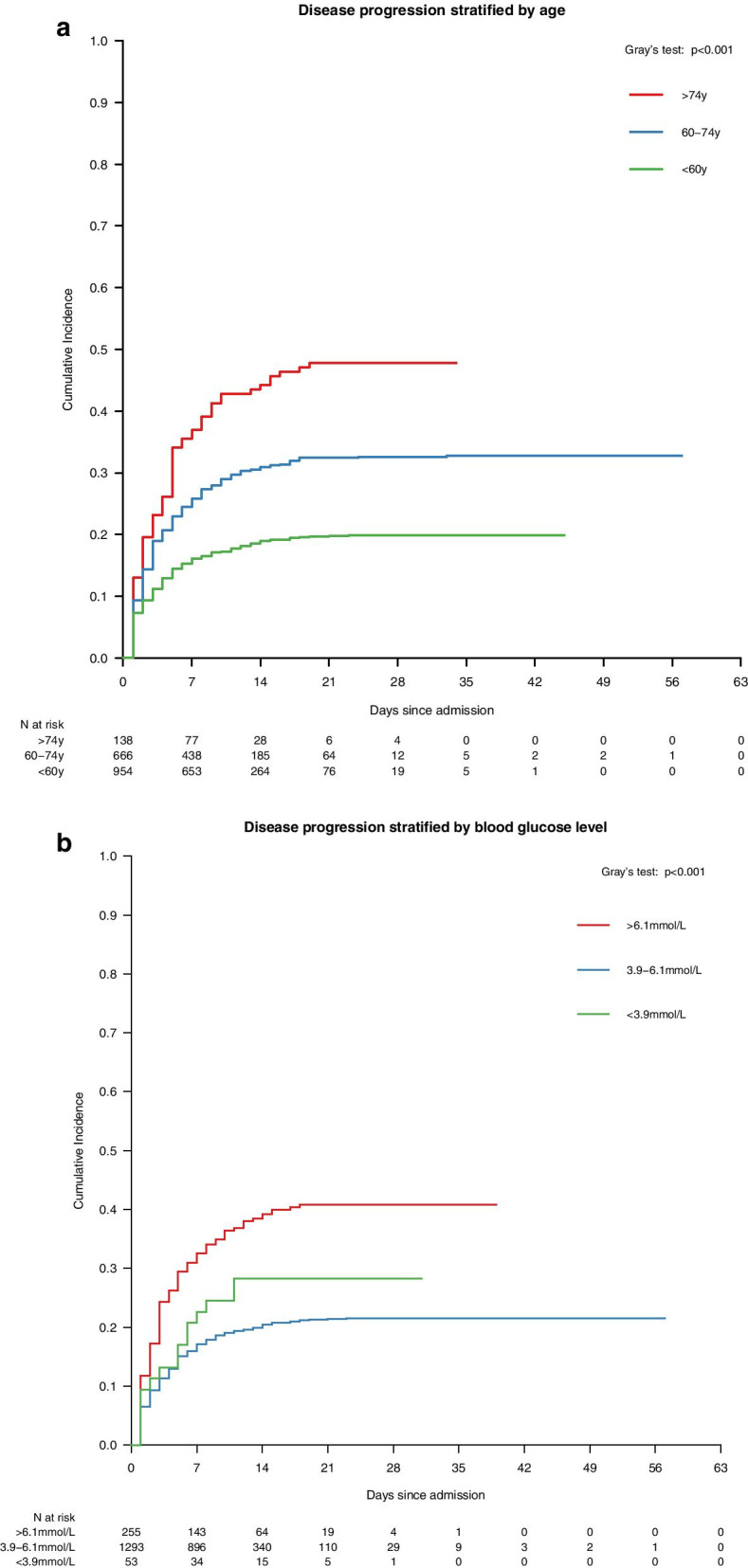

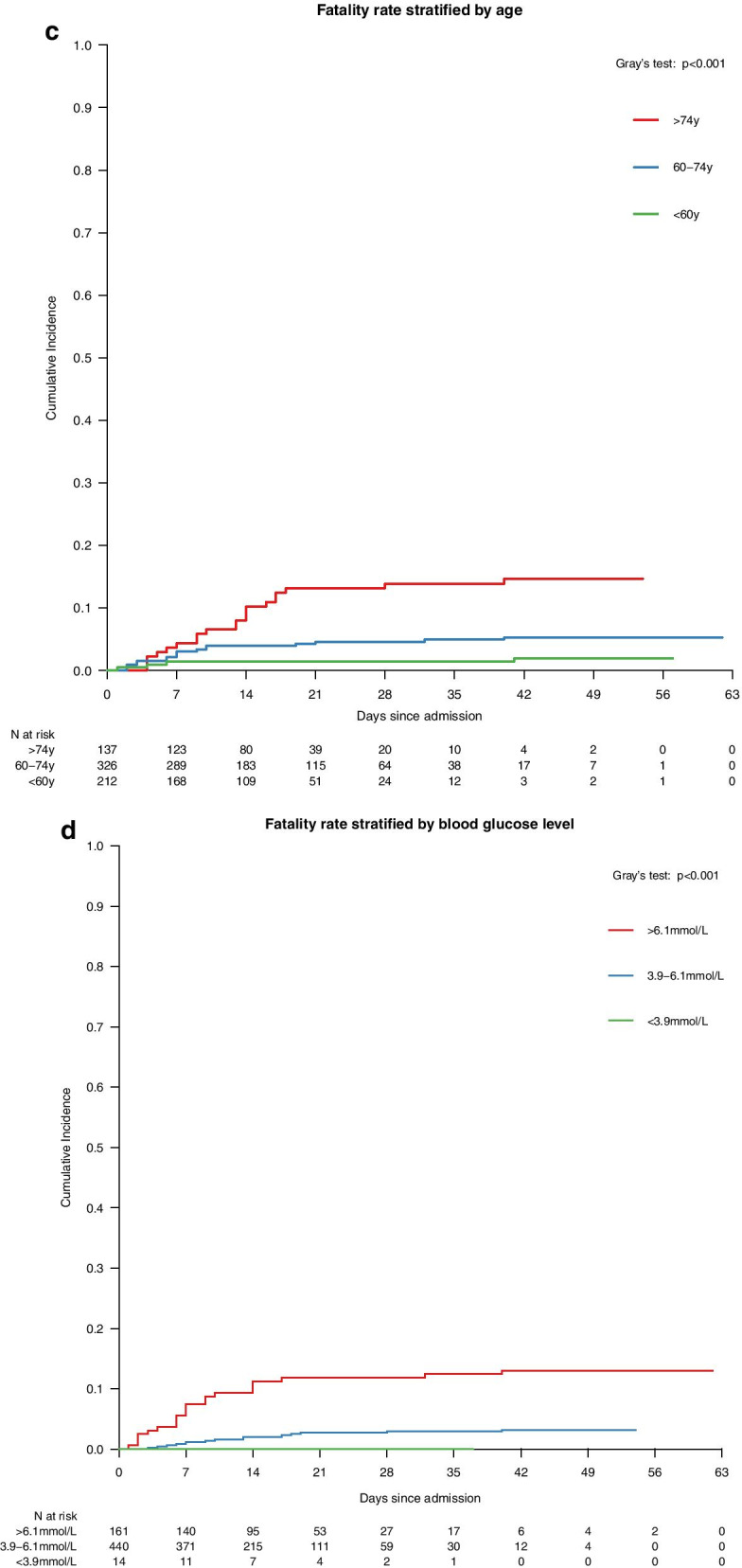
Survival curves showing the cumulative incidence of the first disease progression to severe/critical stages, stratified by (**a**) age, (**b**) blood glucose level; and disease fatality stratified by (**c**) age and (**d**) blood glucose level in 2,433 COVID-19 patients at the Huoshenshan hospital

Of 1,758 mild and moderate patients at admission, 474 (27.0%) progressed to severe or critical severity during hospitalization. Multi-variable cause-specific Cox proportional hazard model (Table 2) identified that patients with age 60–74 years (HR = 1.26, 95%CI 1.02–1.56), > 74 years (1.44, 1.02–2.03), respiratory rate > 20 times/min (1.28, 1.05–1.57), fever (temperature ≥ 37.5 °C) (1.93, 1.21–3.08), chest tightness (1.47, 1.12–1.92), blood glucose > 6.1 mmol/L (1.58, 1.25–1.98), c-reaction protein > 4 mg/L (1.45, 1.12–1.87), lactate dehydrogenase > 250 IU/L (1.63, 1.20–2.20), direct bilirubin > 8 μmol/L (1.51, 1.03, 2.21), albumin < 40 g/L (1.38, 1.07–1.77) and lymphocyte count < 1.1*10^9^/L (1.44, 1.15–1.81) were risk factors for disease progression to severe and critical stage (Table 2). The 21-day cumulative incidence of progression was 47.8% in > 74-years age group, followed by 32.4%, 19.8% in age groups of 60–74 and < 60-years respectively. The cumulative incidence of disease progression at day 21 was also much higher in patients with blood glucose ≥ 6.1 mmol/L (40.8%) than blood glucose in range of 3.9–6.1 mmol/L (21.4%, Fig. 3a, b).

**Table 2:** Multivariate cox proportional hazards regression for disease progression and fatality among COVID-19 patients in Huoshenshan hospital

Variable	The first disease progression from mild/moderate at admission to severe/critical during hospitalization		Disease fatality among patients with severe/critical at admission during hospitalization	
	HR (95% CI)	*p* value	HR (95% CI)	*P* value
*Demographic characteristics*				
Age (year)				
< 60	Reference		Reference	
60–74	1.26 (1.02, 1.56)	0.033*	1.46 (0.47, 4.58)	0.511
> 74	1.44 (1.02, 2.03)	0.037*	3.41 (1.07, 10.89)	0.038*
Respiratory rate > 20 (%)	1.28 (1.05, 1.57)	0.015*		
*Signs and symptoms—no. (%)*				
Fever (temperature ≥ 37.5 °C)	1.93 (1.21, 3.08)	0.007*		
Chest tightness	1.47 (1.12, 1.92)	0.005*		
Fatigue	1.14 (0.94, 1.37)	0.169		
*Laboratory findings*				
Blood glucose (mmol/L)				
3.9–6.1	Reference		Reference	
< 3.9	1.65 (0.97, 2.81)	0.065	7.31 (0.00, inf)	0.996
> 6.1	1.58 (1.25, 1.98)	< 0.001**	3.22 (1.54, 6.73)	0.002*
C-reactive protein (mg/L)				
≤ 4	Reference			
> 4	1.45 (1.12, 1.87)	0.004*		
D-dimer (mg/L)				
≤ 0.55	Reference			
> 0.55	1.27 (0.96, 1.67)	0.099		
Lymphocyte count (10^9^/L)				
1.1–3.2	reference			
< 1.1	1.44 (1.15, 1.81)	0.002*		
> 3.2	0.87 (0.31, 2.40)	0.784		
Lactate dehydrogenase (IU/L)				
120–250	Reference			
< 120	1.38 (0.78, 2.42)	0.267		
> 250	1.63 (1.20, 2.20)	< 0.001**		
Direct bilirubin (μmol/L)				
≤ 8	Reference			
> 8	1.51 (1.03, 2.21)	0.035		
Platelets count (10^9^/L)				
125–350	Reference		Reference	
< 125	0.65 (0.41, 1.05)	0.078	4.39 (2.02, 9.54)	< 0.001**
> 350	1.12 (0.82, 1.53)	0.480	2.60 (0.56, 11.92)	0.220
Fibrinogen (g/L)				
2–4	Reference		Reference	
< 2	1.16 (0.53, 2.53)	0.718	6.48 (1.46, 28.67)	0.016*
> 4	1.19 (0.89, 1.59)	0.799	0.77 (0.29, 2.09)	0.611
Monocyte count (10^9^/L)				
0.1–0.6	Reference			
< 0.1	0.32 (0.07, 1.37)	0.125		
> 0.6	0.96 (0.72, 1.27)	0.762		
Albumin (g/L)				
40–55	Reference			
< 40	1.38 (1.07, 1.77)	0.013*		
> 55	0.0009 (0.00, inf)	0.992		
Aspartate aminotransferase (IU/L)				
≤ 40	Reference			
> 40	1.24 (0.90, 1.72)	0.189		
Neutrophils count (10^9^/L)				
1.8–6.3	Reference			
< 1.8	1.24 (0.81, 1.88)	0.323		
> 6.3	1.22 (0.87, 1.71)	0.244		
Urea nitrogen (mmol/L)				
2.9–7.5	Reference			
< 2.9	0.83 (0.59, 1.17)	0.277		
> 7.5	0.99 (0.64, 1.53)	0.964		
Cystatin C (mg/L)				
0.51–1.09	Reference			
< 0.51	1.37 (0.28, 7.00)	0.696		
> 1.09	1.01 (0.77, 1.31)	0.980		
Creatine kinase-MB (IU/L)				
≤ 24			Reference	
> 24			6.29 (2.51, 15.80)	< 0.001**

^*^Compared with the above reference, the *p* value is between 0.05 and 0.001; **Compared with the above reference, the *p* value < 0.001

Of 675 severe or critical patients at admission, 634 (93.9%) were discharged and 41 (6.1%) died during hospitalization. Table 2 showed that patients with age > 74 years (3.41, 1.07–10.89), blood glucose > 6.1 mmol/L (3.22, 1.54–6.73), platelets count < 125*10^9^/L (4.39, 2.02–9.54), fibrinogen < 2 g/L (6.48, 1.46–28.67) and creatine kinase-MB > 24 IU/L (6.29, 2.51–15.80) were risk factors for in-hospital fatality (Table 2). The 21-day cumulative incidence of fatality was 13.1% in > 74 years age group, 4.6%, 1.4% in age groups of 60–74, < 60 years respectively. The incidence of fatality at day 21 was four times higher in patients with blood glucose > 6.1 g/L (11.8%) than blood glucose in range of 3.9–6.1 mmol/L (2.7%, Fig. 3c, d).

## Discussion

Our study provides unique progression and outcome data on a cohort of 2,433 COVID-19 patients admitted to Huoshenshan hospital, a hospital designed and built solely to provide care to patients with COVID-19. Our findings suggest that even among inpatients with moderately severe disease, the fatality was relatively low. Most deaths arose from patients who were critically ill on admission or progressed to being critical during admission. The risk factors we identified for death and disease progression are similar to previous studies with older age, poor systematic immune and inflammatory responses, and multiple organ damages [14–16, 18, 24–32]. Patients admitted with a greater disease severity requires longer to recover.

Our report on the time for disease progression at each disease stages allows early preparation and intervention to delay disease progression (Fig. 2). Since there is no effective cure for COVID-19, delaying the progression of the disease is key for survival. Most mild or moderate patients (73.0%) did not progress to severe or critical states and recovered from COVID-19 infection in 10.0 (7.0–15.0) days. But among those who did progress, they progressed to a severe or critical stage within the first 3.0 (1.0–7.0) days after admission. This highlights the importance of close monitoring of key risk indicators for disease progression in the early stages of infection. Because, once a patient reaches the critical stage, the chance of fatality is 62.5% in a short period of 6.5 (2.0–13.3) days.

We identified the blood glucose level to be an important prognostic predictor for both COVID-19 progression and fatality. In particular, patients with an elevated level of blood glucose > 6.1 mmol/L had a 58% higher risk of disease progression and 3.22-fold higher risk of fatality. This is consistent with previous reports that a high glucose level contributes to the development of acute respiratory distress syndrome in COVID-19 patients [16, 17, 28, 29]. Uncontrolled blood glucose level also substantially contributes to other comorbidities, including atherosclerosis, diabetic nephropathy, peripheral arteriosclerosis, and diabetic ketoacidosis, which are also causes of COVID-19 related fatality [28, 33]. The underlying mechanism of how blood glucose interacts with SARS-COV-2 is currently unclear. SARS-COV-2 relies on the binding to membrane angiotensin-converting enzyme-2 (ACE-2) receptor to enter pulmonary cells in the lungs. We speculate that diabetic patients have elevated expression of angiotensin-converting enzyme-2 (ACE-2) receptors, making them vulnerable to SARS-COV-2 infection. Besides, patients living with diabetes or uncontrolled glucose level are likely to have impaired innate immunity due to dysfunction of macrophage and lymphocytes, which may lead to an increased risk of septic shock and multiple organ failures. Hyperglycemia is also a potential indicator of pancreatic islet cell injury [33]. Effective glycemic monitor and management may be beneficial to reduce the progression and fatality of COVID-19 patients [34, 35].

Our study reported important clinical indicators that are associated with COVID-19 disease progression and fatality. At the systemic level, a low lymphocyte count is an indication of a poor systematic immune response against the infection, whereas a high c-reactive protein level is an early indicator of systemic inflammatory response syndrome [36]. Besides, increased fibrinogen and platelets count concentrations are associated with increased coagulation activity in patients with infection or sepsis [37, 38]. This is particularly life-threatening for patients with pre-existing cerebro-or cardiovascular diseases, which is common among the elderly. In addition, an elevated creatine kinase-MB level, a know indicator for acute myocardial injury. Further, liver damage or dysfunction, marked by reduced albumin level and elevated direct bilirubin [39]. Most of the remaining indicators may be associated with multiple organ injury or failure. Lactate dehydrogenase predicts the severity of tissue damage. As lactate dehydrogenase is largely present in lung tissues, when damaged by SARS-COV-2 infection, a large amount of lactate dehydrogenase into the circulation. This often clinically presents as a severe form of interstitial pneumonia and subsequently evolve into acute respiratory distress syndrome. Elevated lactate dehydrogenase level is also a predictor of endothelial damage, which caused microvascular thrombosis and associated with renal failure [40]. Maintaining an effective systemic immune response against the infection and prevention of multiple organ failures are, therefore, the priorities in treating COVID-19 patients.

Our study has several limitations. First, this is a retrospective single-centre study, and almost half of patients did not have laboratory tests for interleukin-6, natriuretic peptide type B, supersensitive troponin I, myoglobin and procalcitonin levels within the first day after admission. Their role may be underestimated in the prediction of disease progression and fatality. Second, most patients on admission had moderate severity which may lead to a selection bias when identifying factors that affect progression or fatality. Additional cohort studies of patients with COVID-19 pneumonia from areas outside Wuhan are needed to confirm our results. Third, considering both the small number of events and the rule of thumb on event per variable > 10, only limited risk factors were included in the multivariable analysis of severe and critically illness at admission to fatality during hospitalization. Fourth, most patients were referred from other medical institution, and the data at diagnosis was not accessible to us.

## Conclusions

In this study, we comprehensively presented stage-wise disease’s first progression among COVID-19 patients. We identified that older age, elevated glucose level, together with other clinical indicators associated with systemic responses and multiple organ failures, predicted both the disease progression and fatality among COVID-19 patients.

## Supplementary Information

**Additional file 1.** Statistical analysis for key indicators of COVID-19 progression, fatality, and associated factors.

## Data Availability

The data that support the findings of this study are available from the Huoshenshan hospital but restrictions apply to the availability of these data, which were used under license for the current study, and so are not publicly available. Data are however available from the authors upon reasonable request and with permission of the Huoshenshan hospital.
