# Supplementary material for: Elevated glucose level leads to rapid COVID-19 progression and high fatality
Source: BMC Pulm Med. 2021 Feb 24;21:64. doi: 10.1186/s12890-021-01413-w (PMC7903375; doi:10.1186/s12890-021-01413-w)
Supplement: Supplementary file 1 — Additional file 1. Statistical analysis for key indicators of COVID-19 progression, fatality, and associated factors. [file 12890_2021_1413_MOESM1_ESM.doc]

**Supplemental Appendix**

**Elevated glucose level leads to rapid COVID-19 progression and high fatality**

Wenjun Wang^1,2,3^*, Mingwang Shen^4^*, Yusha Tao^4^, Christopher K Fairley^4,5^, Qin Zhong^1,2,3^, Zongren Li^1,2,3^, Hui Chen^6,7^, Jason J Ong^4,5^, Dawei Zhang^8^, Kai Zhang^1^, Ning Xing^10^, Huayuan Guo^1^, Enqiang Qin^8^, Xizhou Guan^11^, Feifei Yang^1^, Sibing Zhang^6,9^, Lei Zhang^4,5⁋^, Kunlun He^1,2,3⁋^

1. Key Laboratory of Ministry of Industry and Information Technology of Biomedical Engineering and Translational Medicine, Chinese PLA General Hospital, Beijing, 100853, P.R.China.
2. Translational Medical Research Center, Chinese PLA General Hospital, Beijing, 100853, P.R.China.
3. Medical Artificial Intelligence Research Center, Chinese PLA General Hospital, Beijing, 100853, P.R.China.
4. China-Australia Joint Research Center for Infectious Diseases, School of Public Health, Xi’an Jiaotong University Health Science Center, Xi’an, Shanxi, 710061, PR China.
5. Central Clinical School, Faculty of Medicine, Nursing and Health Sciences, Monash University, Melbourne, VIC, Australia.
6. Department of Medical Information, Huoshenshan Hospital, Wuhan Hubei, China.
7. Department of Medical Information, the 940th Hospital of PLA Joint Logistics Support Force, Lanzhou, China.
8. Department of Infectious Disease, the Fifth Medical Center, Chinese PLA General Hospital, Beijing, 100039, P.R.China.
9. Department of Medical Administration, Chinese PLA General Hospital, Beijing, 100853, P.R.China.
10. Department of radiology, Chinese PLA General Hospital, Beijing, 100853, P.R.China.
11. Department of Pulmonary and Critical Care Medicine, Chinese PLA General Hospital, Beijing, 100853, P.R.China.

*authors contribute equally

^⁋^authors contribute to supervision equally

Corresponding authors:

- Kunlun He, [**kunlunhe@plagh.org**](mailto:hekl301@aliyun.com);
- Lei Zhang, [**lei.zhang1@xjtu.edu.cn**](mailto:lei.zhang1@xjtu.edu.cn)

**Definition of clinical complications**

1. Cough, Fatigue, Diarrhea, Chest tightness and Shortness of breath symptoms of pneumonia were defined in accordance with the American Lung Association[1].
2. Septic shock was defined as a subset of sepsis in which particularly profound circulatory, cellular, and metabolic abnormalities are associated with a greater risk of mortality than with sepsis alone[2].

**Reference**

**Table S1. Complete Univariate and Multivariate cox proportional hazards regressions for disease progression and fatality among COVID-19 patients in Huoshenshan hospital, China (2020)**

| **Variable** | The first disease progression from mild/moderate  severity at admission to severe/critical severity during hospitalization | | | | |  | Disease fatality among patients with severe/critical  severity at admission during hospitalization | | | | | |
| --- | --- | --- | --- | --- | --- | --- | --- | --- | --- | --- | --- | --- |
|  |  |  |  |  |  |  |  |  |  |  |  |  |
|  | Univariate Cox Regression | |  | Multivariate Cox regression | |  | Univariate Cox Regression | |  | Multivariate Cox regression | |  |
|  | HR (95% CI) | P-value |  | HR (95% CI) | P-value |  | HR (95% CI) | P-value |  | HR (95% CI) | P-value |  |
| **Demographic characteristics** | | | | | | | | | | | | |
| Age (yr) |  |  |  |  |  |  |  |  |  |  |  | |
| <60 | reference |  |  | reference |  |  | reference |  |  | reference |  | |
| 60, 74 | 1.70 (1.40, 2.06) | <0.001** |  | 1.26 (1.02, 1.56) | 0.033* |  | 2.39 (0.78, 6.93) | 0.13 |  | 1.46 (0.47, 4.58) | 0.511 | |
| >74 | 2.74 (2.07, 3.63) | <0.001** |  | 1.44 (1.02, 2.03) | 0.037* |  | 6.27 (2.33, 19.96) | <0.001** |  | 3.41 (1.07, 10.89) | 0.038* | |
| Male gender | 1.17 (0.97, 1.40) | 0.094 |  |  |  |  | 0.54 (0.30, 0.11) | 0.094 |  |  |  | |
| Current smoking | 1.22 (0.90, 1.65) | 0.206 |  |  |  |  | 1.90 (0.74, 4.22) | 0.198 |  |  |  | |
| Current drinking | 1.21 (0.86, 1.69) | 0.274 |  |  |  |  | 1.49 (0.41, 4.33) | 0.632 |  |  |  | |
| Respiratory rate >20 (%) | 1.43 (1.18, 1.74) | <0.001** |  | 1.28 (1.05, 1.57) | 0.015* |  | 2.28 (1.32, 4.63) | 0.005* |  |  |  | |
| Pulse rate >100 (%) | 0.80 (0.60, 1.07) | 0.131 |  |  |  |  | 1.57 (0.81, 3.37) | 0.170 |  |  |  | |
| Systolic blood pressure ≥140 (mmHg) | 1.11 (0.89, 1.35) | 0.379 |  |  |  |  | 0.88 (0.45, 1.79) | 0.751 |  |  |  | |
| Diastolic blood pressure ≥90 (mmHg) | 0.87 (0.70, 1.09) | 0.226 |  |  |  |  | 0.20 (0.05, 0.89) | 0.034* |  |  |  | |
| **Signs and Symptoms — no. (%)** |  |  |  |  |  |  |  |  |  |  |  | |
| Fever (temperature≥37.5℃) | 4.11 (2.72, 6.20) | <0.001** |  | 1.93 (1.21, 3.08) | 0.007* |  | 3.32 (1.43, 7.44) | 0.005* |  |  |  | |
| Cough | 0.97 (0.81, 1.16) | 0.724 |  |  |  |  | 0.53 (0.23, 1.81) | 0.280 |  |  |  | |
| Fatigue | 1.35 (1.13, 1.61) | 0.001* |  | 1.14 (0.94, 1.37) | 0.169 |  | 1.00 (0.56, 1.92) | 0.918 |  |  |  | |
| Diarrhea | 0.65 (0.34, 1.26) | 0.203 |  |  |  |  | 2.03 (0.55, 9.58) | 0.254 |  |  |  | |
| Chest tightness | 1.41 (1.09, 1.83) | 0.010* |  | 1.47 (1.12, 1.92) | 0.005* |  | 0.72 (0.20, 1.57) | 0.267 |  |  |  | |
| Shortness of breath | 1.27 (1.04, 1.55) | 0.019* |  |  |  |  | 0.75 (0.39, 1.56) | 0.478 |  |  |  | |
| **Comorbidities** |  |  |  |  |  |  |  |  |  |  |  | |
| Hypertension | 1.31 (1.08, 1.59) | 0.005* |  |  |  |  | 0.55 (0.30, 1.15) | 0.120 |  |  |  | |
| Diabetes | 1.26 (0.99, 1.62) | 0.065 |  |  |  |  | 0.74 (0.32, 1.83) | 0.553 |  |  |  | |
| Coronary heart disease | 1.43 (1.02, 2.01) | 0.040* |  |  |  |  | 2.21 (1.10, 5.19) | 0.033* |  |  |  | |
| Cancer | 0.85 (0.40, 1.79) | 0.661 |  |  |  |  | 0.89 (0.14, 7.42) | 0.904 |  |  |  | |
| Chronic bronchitis | 0.84 (0.40, 1.78) | 0.651 |  |  |  |  | 1.10 (0.31, 5.45) | 0.895 |  |  |  | |
| Cerebrovascular disease | 1.40 (0.85, 2.30) | 0.187 |  |  |  |  | 0.77 (0.21, 3.54) | 0.721 |  |  |  | |
| Chronic kidney disease | 0.76 (0.31, 1.83) | 0.542 |  |  |  |  | 3.83 (1.42, 11.21) | 0.010* |  |  |  | |
| Chronic obstructive pulmonary disease | 1.48 (0.48, 4.62) | 0.495 |  |  |  |  | 0.00 (0.00, inf) | 0.996 |  |  |  | |
| Hepatitis | 0.99 (0.41, 2.39s) | 0.983 |  |  |  |  | 0.00 (0.00, inf) | 0.996 |  |  |  | |
| **Laboratory findings** | | | | | | | | | | | | |
| Blood glucose (mmol/L) |  |  |  |  |  |  |  |  |  |  |  | |
| 3.9-6.1 | reference |  |  | reference |  |  | reference |  |  | reference |  | |
| <3.9 | 1.34 (0.80, 2.26) | 0.265 |  | 1.65 (0.97, 2.81) | 0.065 |  | 0.00 (0.00, inf) | 0.997 |  | 7.31 (0.00, inf) | 0.996 | |
| >6.1 | 2.10 (1.68, 2.63) | <0.001** |  | 1.58 (1.25, 1.98) | <0.001** |  | 3.28 (1.87, 7.27) | <0.001** |  | 3.22 (1.54, 6.73) | 0.002* | |
| C-reactive protein (mg/L) |  |  |  |  |  |  |  |  |  |  |  | |
| ≤4 | reference |  |  | reference |  |  | reference |  |  |  |  | |
| >4 | 2.42 (1.98, 2.97) | <0.001** |  | 1.45 (1.12, 1.87) | 0.004* |  | 19.97 (2.71, 147.07) | 0.003* |  |  |  | |
| D-dimer (mg/L) |  |  |  |  |  |  |  |  |  |  |  | |
| ≤0.55 | reference |  |  | reference |  |  | reference |  |  |  |  | |
| >0.55 | 2.27 (1.82, 2.84) | <0.001** |  | 1.27 (0.96, 1.67) | 0.099 |  | 3.56 (1.40, 15.94) | 0.020* |  |  |  | |
| Lymphocyte count (10^9^/L) |  |  |  |  |  |  |  |  |  |  |  | |
| 1.1-3.2 | reference |  |  | reference |  |  | reference |  |  |  |  | |
| <1.1 | 2.40 (1.98, 2.91) | <0.001** |  | 1.44 (1.15, 1.81) | 0.002* |  | 7.54 (3.23, 21.50) | <0.001** |  |  |  | |
| >3.2 | 0.96 (0.36, 2.59) | 0.943 |  | 0.87 (0.31, 2.40) | 0.784 |  | 0.00 (0.00, inf) | 0.997 |  |  |  | |
| Lactate dehydrogenase (IU/L) |  |  |  |  |  |  |  |  |  |  |  | |
| 120-250 | reference |  |  | reference |  |  | reference |  |  |  |  | |
| <120 | 1.06 (0.62, 1.81) | 0.844 |  | 1.38 (0.78, 2.42) | 0.267 |  | 0.00 (0.00, inf) | 0.998 |  |  |  | |
| >250 | 3.31 (2.59, 4.23) | <0.001** |  | 1.63 (1.20, 2.20) | <0.001** |  | 14.38 (4.86, 40.57) | <0.001** |  |  |  | |
| Direct bilirubin (umol/L) |  |  |  |  |  |  |  |  |  |  |  | |
| ≤8 | reference |  |  | reference |  |  | reference |  |  |  |  | |
| >8 | 2.44 (1.67, 3.57) | <0.001** |  | 1.51 (1.03, 2.21) | 0.035 |  | 5.27 (3.23, 14.84) | <0.001** |  |  |  | |
| Platelets count (10^9^/L) |  |  |  |  |  |  |  |  |  |  |  | |
| 125-350 | reference |  |  | reference |  |  | reference |  |  | reference |  | |
| <125 | 1.06 (0.67, 1.66) | 0.812 |  | 0.65 (0.41, 1.05) | 0.078 |  | 7.63 (4.86, 18.09) | <0.001** |  | 4.39 (2.02, 9.54) | <0.001** | |
| >350 | 1.47 (1.11, 1.97) | 0.008* |  | 1.12 (0.82, 1.53) | 0.480 |  | 1.19 (0.45, 8.43) | 0.377 |  | 2.60 (0.56, 11.92) | 0.220 | |
| Fibrinogen (g/L ) |  |  |  |  |  |  |  |  |  |  |  | |
| 2-4 | reference |  |  | reference |  |  | reference |  |  | reference |  | |
| <2 | 0.95 (0.35, 2.56) | 0.923 |  | 1.16 (0.53, 2.53) | 0.718 |  | 22.21 (6.39, 80.97) | <0.001** |  | 6.48 (1.46, 28.67) | 0.016* | |
| >4 | 2.70 (1.98, 3.69) | <0.001** |  | 1.19 (0.89, 1.59) | 0.799 |  | 1.04 (0.32, 3.08) | 0.992 |  | 0.77 (0.29, 2.09) | 0.611 | |
| Monocyte count (10^9^/L) |  |  |  |  |  |  |  |  |  |  |  | |
| 0.1-0.6 | reference |  |  | reference |  |  | reference |  |  |  |  | |
| <0.1 | 1.27 (0.32, 5.08) | 0.740 |  | 0.32 (0.07, 1.37) | 0.125 |  | 1.52 (0.32, 17.27) | 0.402 |  |  |  | |
| >0.6 | 1.30 (1.01, 1.66) | 0.040* |  | 0.96 (0.72, 1.27) | 0.762 |  | 0.89 (0.28, 1.61) | 0.370 |  |  |  | |
| Albumin (g/L) |  |  |  |  |  |  |  |  |  |  |  | |
| 40-55 | reference |  |  | reference |  |  | reference |  |  |  |  | |
| <40 | 2.06 (1.62, 2.61) | <0.001** |  | 1.38 (1.07, 1.77) | 0.013* |  | 5.28 (0.72, 38.25) | 0.107 |  |  |  | |
| >55 | 0.00 (0.00, inf) | 0.991 |  | 0.0009 (0.00, inf) | 0.992 |  |  |  |  |  |  | |
| Aspartate aminotransferase (IU/L) |  |  |  |  |  |  |  |  |  |  |  | |
| ≤40 | reference |  |  | reference |  |  | reference |  |  |  |  | |
| >40 | 1.89 (1.42, 2.51) | <0.001** |  | 1.24 (0.90, 1.72) | 0.189 |  | 3.68 (2.10, 9.08) | <0.001** |  |  |  | |
| Neutrophils count (10^9^/L) |  |  |  |  |  |  |  |  |  |  |  | |
| 1.8-6.3 | reference |  |  | reference |  |  | reference |  |  |  |  | |
| <1.8 | 1.25 (0.84, 1.87) | 0.263 |  | 1.24 (0.81, 1.88) | 0.323 |  | 0.70 (0.08, 4.97) | 0.674 |  |  |  | |
| >6.3 | 2.15 (1.61, 2.86) | <0.001** |  | 1.22 (0.87, 1.71) | 0.244 |  | 4.80 (0.65, 35.63) | 0.125 |  |  |  | |
| Urea nitrogen (mmol/L) |  |  |  |  |  |  |  |  |  |  |  | |
| 2.9-7.5 | reference |  |  | reference |  |  | reference |  |  |  |  | |
| <2.9 | 0.86 (0.62, 1.21) | 0.386 |  | 0.83 (0.59, 1.17) | 0.277 |  | 1.22 (0.09, 5.21) | 0.715 |  |  |  | |
| >7.5 | 1.76 (1.18, 2.62) | 0.006* |  | 0.99 (0.64, 1.53) | 0.964 |  | 6.97 (3.57, 14.60) | <0.001** |  |  |  | |
| Cystatin C (mg/L) |  |  |  |  |  |  |  |  |  |  |  | |
| 0.51-1.09 | reference |  |  | reference |  |  | reference |  |  |  |  | |
| <0.51 | 1.13 (0.16, 8.08) | 0.900 |  | 1.37 (0.28, 7.00) | 0.696 |  | 0.00 (0.00, inf) | 0.997 |  |  |  | |
| >1.09 | 1.79 (1.42, 2.25) | <0.001** |  | 1.01 (0.77, 1.31) | 0.980 |  | 2.76 (1.64, 6.65) | 0.001* |  |  |  | |
| Creatine kinase-MB (IU/L) |  |  |  |  |  |  |  |  |  |  |  | |
| ≤24 | reference |  |  |  |  |  | reference |  |  | reference |  | |
| >24 | 1.88 (1.06, 3.35) | 0.031 |  |  |  |  | 8.56 (4.95, 23.65) | <0.001** |  | 6.29 (2.51, 15.80) | <0.001** | |
| Prothrombin time (s) |  |  |  |  |  |  |  |  |  |  |  | |
| 9.2-15 | reference |  |  |  |  |  | reference |  |  |  |  | |
| >15 | 1.45 (0.88, 2.39) | 0.149 |  |  |  |  | 10.96 (5.05, 25.30) | <0.001** |  |  |  | |
| Basophil count (10^8^/L) |  |  |  |  |  |  |  |  |  |  |  | |
| 0-0.06 | reference |  |  |  |  |  | reference |  |  |  |  | |
| >0.06 | 0.70 (0.33, 1.47) | 0.346 |  |  |  |  | 0.00 (0.00, inf) | 0.996 |  |  |  | |
| Eosinophil count (10^8^/L) |  |  |  |  |  |  |  |  |  |  |  | |
| 0.02-0.52 | reference |  |  |  |  |  | reference |  |  |  |  | |
| <0.02 | 2.36 (1.80, 3.10) | <0.001** |  |  |  |  | 4.97 (3.21, 11.96) | <0.001** |  |  |  | |
| >0.52 | 1.26 (0.60, 2.67) | 0.542 |  |  |  |  | 2.45 (0.43, 24.52) | 0.254 |  |  |  | |
| Total bilirubin (*μ*mol/L) |  |  |  |  |  |  |  |  |  |  |  | |
| 5.13-22.24 | reference |  |  |  |  |  | reference |  |  |  |  | |
| >22.24 | 1.43 (0.89, 2.30) | 0.138 |  |  |  |  | 5.11 (3.23, 14.84) | <0.001** |  |  |  | |
| White blood cell count (10^9^/L) |  |  |  |  |  |  |  |  |  |  |  | |
| 4-10 | reference |  |  |  |  |  | reference |  |  |  |  | |
| <4 | 1.14 (0.87, 1.51) | 0.344 |  |  |  |  | 1.57 (0.37, 4.28) | 0.712 |  |  |  | |
| >10 | 1.84 (1.24, 2.72) | 0.002* |  |  |  |  | 7.81 (3.58, 13.74) | <0.001** |  |  |  | |
| Alkaline phosphatase (IU/L) |  |  |  |  |  |  |  |  |  |  |  | |
| 45-135 | reference |  |  |  |  |  | reference |  |  |  |  | |
| <45 | 1.03 (0.65, 1.63) | 0.915 |  |  |  |  | 0.80 (0.00, inf) | 0.997 |  |  |  | |
| >135 | 1.50 (0.94, 2.38) | 0.087 |  |  |  |  | 4.28 (2.21, 12.17) | 0.001* |  |  |  | |
| Creatinine (*μ*mol/L) |  |  |  |  |  |  |  |  |  |  |  | |
| 44-106 | reference |  |  |  |  |  | reference |  |  |  |  | |
| <44 | 1.07 (0.63, 1.83) | 0.800 |  |  |  |  | 0.62 (0.09, 5.16) | 0.639 |  |  |  | |
| >106 | 1.69 (1.02, 2.78) | 0.041* |  |  |  |  | 5.33 (2.80, 13.34) | <0.001** |  |  |  | |
| Creatine kinase (U/L) |  |  |  |  |  |  |  |  |  |  |  | |
| 24-170 | reference |  |  |  |  |  | reference |  |  |  |  | |
| <24 | 1.21 (0.50, 2.93) | 0.668 |  |  |  |  | 0.00 (0.00, inf) | 0.997 |  |  |  | |
| >170 | 2.10 (1.21, 3.66) | 0.009* |  |  |  |  | 3.64 (1.68, 10.25) | 0.002* |  |  |  | |
| Alanine aminotransferase (IU/L) |  |  |  |  |  |  |  |  |  |  |  | |
| 0-40 | reference |  |  |  |  |  | reference |  |  |  |  | |
| >40 | 1.27 (1.01, 1.60) | 0.038* |  |  |  |  | 1.47 (0.85, 3.92) | 0.124 |  |  |  | |

*Compared with the above reference, the P-value is between 0.05 and 0.001; **Compared with the above reference, the P-value < 0.001.

**Figure S1. The least absolute shrinkage and selection operator (LASSO) binary logistic regression model was used to validation selection of mild/moderate disease progression to server/critical stages in 2,433 COVID-19 patients in Huoshenshan hospital (a) LASSO coefficient profiles. (b) Tuning parameter (λ) selection in the LASSO model used 10, fold cross, validation via minimum criteria.**

**
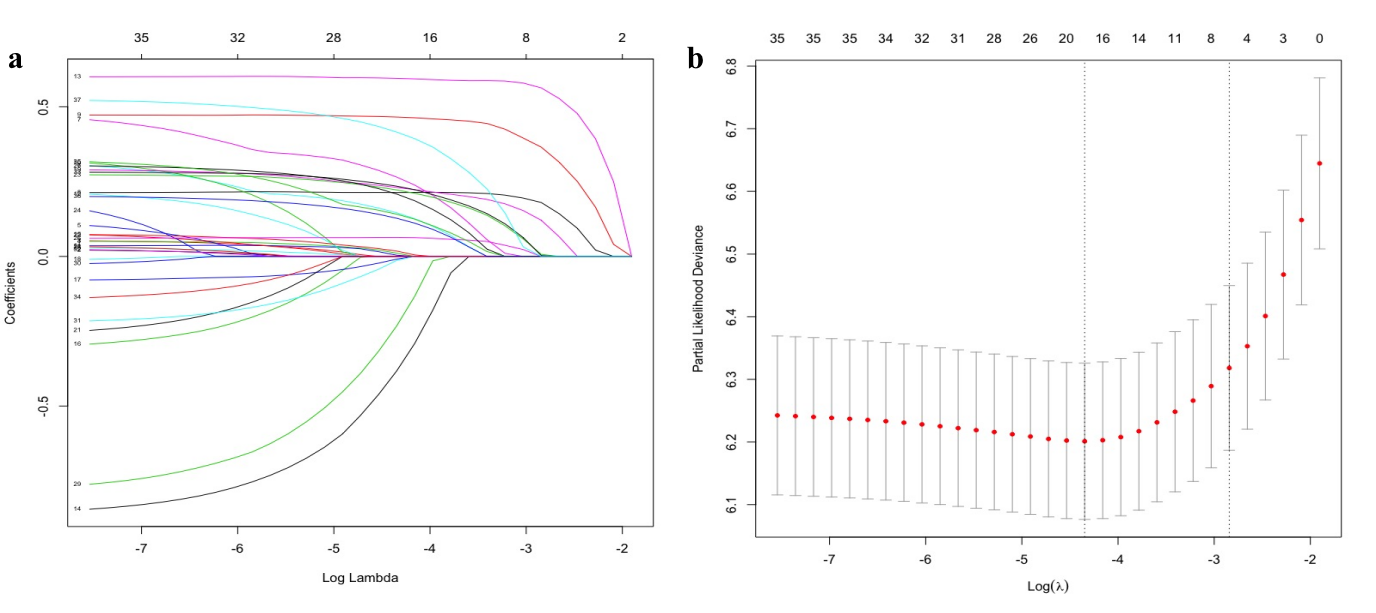
**

**Figure S2. The least absolute shrinkage and selection operator (LASSO) binary logistic regression model was used to validation selection of server/critical disease in, hospital fatality in 2,433 COVID, 19 patients in Huoshenshan hospital (a) LASSO coefficient profiles. (b) Tuning parameter (λ) selection in the LASSO model used 10, fold cross, validation via minimum criteria.**

**
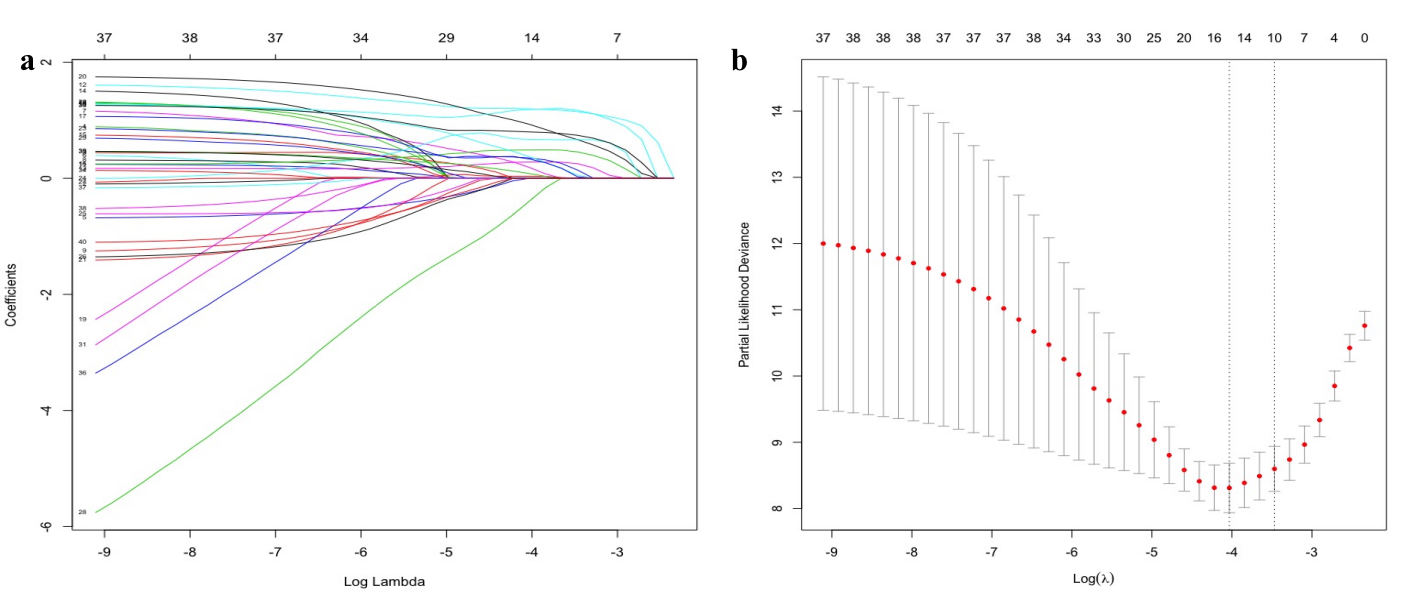
**

**Figure S3. Survival curves that demonstrate the cumulative incidence of disease progression to server/critical stages, stratified by (a) respiratory rate, (b) fever, (c) c-reaction protein, (d) chest tightness, (e) lymphocyte count, (f) lactate dehydrogenase, (g) albumin, (h) direct bilirubin levels in 2,433 COVID-19 patients in Huoshenshan hospital, China (2020).**

**
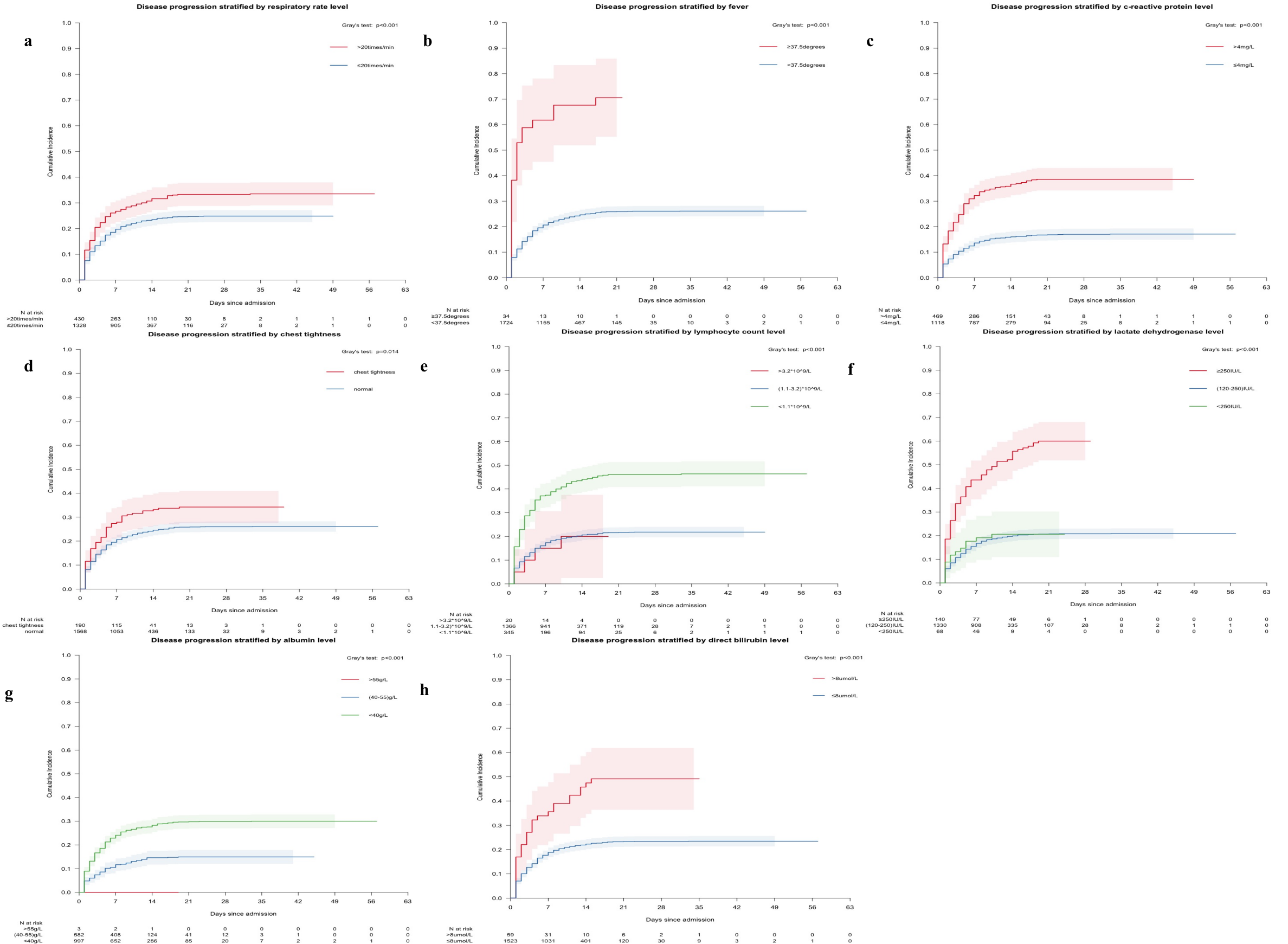
**

**Figure S4. Survival curves that demonstrate the cumulative incidence of disease fatality stratified by (a) platelets count, (b) fibrinogen, (c) creatine kinase-MB level in 2,433 COVID-19 patients in Huoshenshan hospital, China (2020).**

**
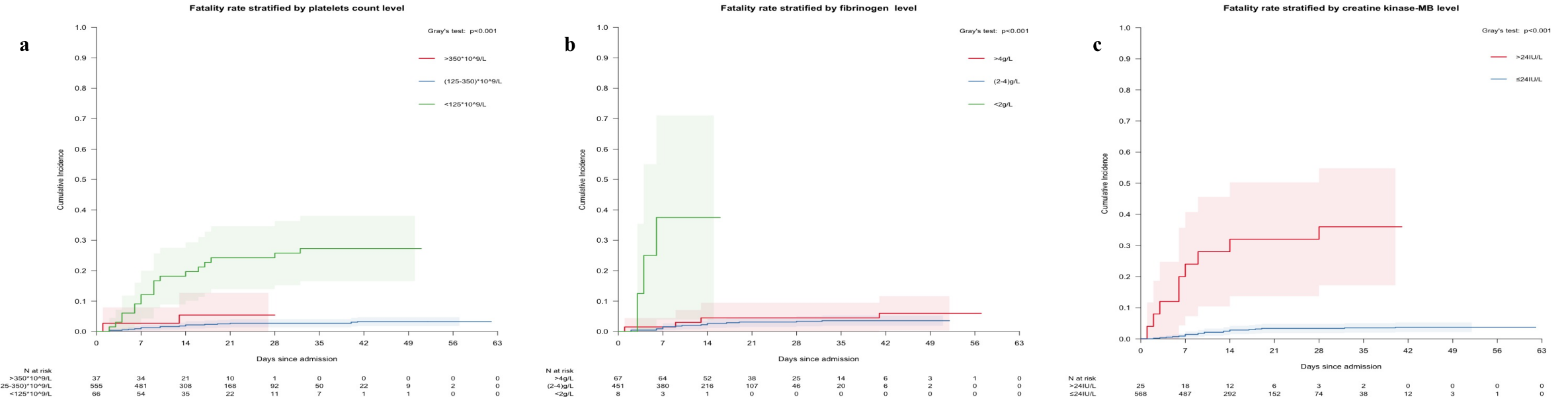
**
